# Electrospun Nisin-Loaded Poly(ε-caprolactone)-Based Active Food Packaging

**DOI:** 10.3390/ma15134540

**Published:** 2022-06-28

**Authors:** Alena Opálková Šišková, Katarína Mosnáčková, Marta Musioł, Andrej Opálek, Mária Bučková, Piotr Rychter, Anita Eckstein Andicsová

**Affiliations:** 1 Polymer Institute of Slovak Academy of Sciences, Dúbravská cesta 9, 845 41 Bratislava, Slovakia; katarina.mosnackova@savba.sk (K.M.); anita.andicsova@savba.sk (A.E.A.); 2 Institute of Materials and Machine Mechanics, Slovak Academy of Sciences, Dúbravská cesta 9, 845 13 Bratislava, Slovakia; andrej.opalek@savba.sk; 3 Centre of Polymer and Carbon Materials, Polish Academy of Sciences, M. Curie-Skłodowska 34, 41-800 Zabrze, Poland; mmusiol@cmpw-pan.edu.pl; 4 Institute of Molecular Biology, Slovak Academy of Sciences, Dúbravská cesta 9, 845 41 Bratislava, Slovakia; maria.buckova@savba.sk; 5 Faculty of Science and Technology, Jan Długosz University in Częstochowa, 13/15 Armii Krajowej Av., 42-200 Częstochowa, Poland; p.rychter@ujd.edu.pl

**Keywords:** poly(ε-caprolactone), nisin, packaging application, electrospun membrane

## Abstract

Packaging for fresh fruits and vegetables with additional properties such as inhibition of pathogens grown can reduce food waste. With its biodegradability, poly(ε-caprolactone) (PCL) is a good candidate for packaging material, especially in the form of an electrospun membrane. The preparation of nonwoven fabric of PCL loaded with food additive, antimicrobial nisin makes them an active packaging with antispoilage properties. During the investigation of the nonwoven fabric mats, different concentrations of nisin were obtained from the solution of PCL via the electrospinning technique. The obtained active porous PCL loaded with varying concentrations of nisin inhibited the growth of *Staphylococcus aureus* and *Escherichia coli*. Packages made of PCL and PCL/nisin fibrous mats demonstrated a prolongation of the fruits’ freshness, improving their shelf life and, consequently, their safety.

## 1. Introduction

In EU countries, the consumption of fresh fruits and vegetables is encouraged to prevent chronic diseases such as heart disease, cancer, diabetes, and obesity [1]. However, since fruits and vegetables are usually consumed raw, they have been recognized as a vehicle to transmit foodborne pathogens [2]. Despite introducing safety practices in production, foodborne outbreaks due to contamination continue to result in illnesses, hospitalizations, and even deaths in many parts of the world [3]. Therefore, food packaging is one of the most important steps in the food industry. A suitable packaging attracts the customer’s attention, keeps the products at the highest possible nutrition level, improves quality, and prolongs their durability [4]. Traditional fossil-based plastic materials, which have been widely used for many decades, have a highly important function in food packaging. However, conventional plastic materials rely on nonrenewable resources, are non-biodegradable, and are not fully recyclable. The massive consumption of such materials contributes to environmental issues such as depleting natural resources, the threat of plastic pollution reflected in the increasing number of illegal dumps, and global warming [5,6]. An increased public awareness of the environmental issues, challenges related to conventional plastic materials, and consumer pressure for improved sustainability have triggered the development of biobased, environmentally friendly food packaging materials. The introduction and implementation of the single-use plastics EU directive, issued by the European Commission in 2020, may accelerate the implementation of alternatives to traditional plastic materials [7,8]. 

Herein, we would like to propose a packaging material playing a doubly beneficial role, namely, not only to be attractive from an environmental protection point of view due to its biodegradability, but also to potentially be used as active packaging. Active packaging offers properties that prolong a food product’s shelf life by preventing aging and spoilage during storage [9]. Following the European regulation (EC) no. 450/2009, “active materials and articles mean materials and articles that are intended to extend the shelf life or to maintain or improve the condition of packaged food; they are designed to deliberately incorporate components that would release or absorb substances into or from the packaged food or the environment surrounding the food”.

The packaging market is still evolving; the functional package replaces conventional ones. Features such as having flavor- or odor-absorbing, moisture-absorbing, and antioxidant-releasing properties have become the norm in food packages. Moreover, biodegradability and antibacterial properties are also often taken into account. Poly(ε-caprolactone) (PCL), as one of the biodegradable alternatives to conventional plastic materials for potential applications in various industrial branches, is being extensively studied for pharmaceutical, biomedical, and tissue engineering applications [10,11]. Polycaprolactone is obtained by the ring-opening polymerization of monomer ε-caprolactone from fossil fuel. This biodegradable polymer is linear and semicrystalline [12]. The glass transition temperature and melting point around this polymer range between −60 and 60 °C, respectively, facilitating its processing but limiting applications. There are a few approaches to applying PCL-based packaging as a film, mainly for biomedical applications [13] and food packaging [14]. However, according to our knowledge, there have been no reports on applying polycaprolactone used as nonwoven mats loaded with an active agent (here antimicrobial Nisin) towards food packaging, strictly fruits and vegetables. Nisin is a heat-stable and nontoxic material that shows broad-spectrum activity against Gram-positive bacterial strains. Still, a high processing temperature for a long time can decrease its antimicrobial ability [15]. It is also on the GRAS list (generally recognized as safe) in the USA, and it has been added to the positive list of food additives (number E 234) in the European Union [16]. The addition of nisin directly into food causes a limitation of the number of bacteria at any given time. However, the bacterial population can recover from a small number of intact cells over time. Hence, the development of food packaging containing nisin appears to be an attractive solution. The possibility of processing at specific temperature ranges without losing the bactericidal capacity is also an added value [17]. 

Nonwoven PCL-based materials are much more popular for antimicrobial and tissue engineering applications [18,19]. It is worth noting that currently, packages of molded fiber using mainly the wood pulp of recycled wastepaper are trendy because they are produced from renewable resources, are totally biodegradable, minimize the volume of packaging waste, and are easily toppled. However, there is no information about using this technique to prepare active packaging, providing a prolongation of fruits’ or vegetables’ freshness. 

Nevertheless, PCL can be successfully applied as a packaging material for fresh fruits and vegetables. The nonwoven fabric covering fabrics from conventional polymers is already used in horticulture and agriculture to protect fruits and vegetables against pests and dust. However, such covers play the role of a physical barrier and are directly used on the shrubs. Moreover, such a material is not biodegradable and may contaminate the environment after use. We intend to design a material and form a nonwoven fabric made of a biodegradable polymer, loaded with a food antispoilage agent meeting the active packaging criteria. 

Electrospinning is an excellent technique that allows the preparation of proposed fibrous bags and creates new possibilities in the packaging area, increasing the sensitivity of packaging to external conditions. This is due to the very large surface area received thanks to obtaining very small fiber diameters. The ambient conditions in which electrospinning occurs allow insertion into the fibers’ thermally labile active agents [20]. Electrospinning allows the functionalization of materials before, during, and after spinning, decreases the cost, enhances commercial availability for industrial production, and fibrous mats can be deposited onto various substrates [21,22]. This technique is a method in which polymers in a solution or melt are formed into continuous fibers by applying an electric field [23]. The drops of polymer solution are squeezed from a syringe, and high electrostatic forces overcome the cohesive forces, which results in the formation of a jet, its continuous elongation in an area of whipping instability, the evaporation of the solvent, and the formation of fibers with diameters in the range from a few nanometers to tens of micrometers [24]. Polycaprolactone is often processed by electrospinning due to the potential to obtain a large specific surface area with a small fiber diameter [25]. 

The main objective of this study was to create an antispoilage nonwoven fabric made of PCL and loaded with nisin as an agent, prolonging the freshness of vegetables and fruits during storage. Such an active packaging will inhibit the activity of common pathogens responsible for the spoilage and prolong the good quality of fresh vegetables before consumption. Therefore, electrospun membranes with different nisin concentrations were prepared from the poly(ε-caprolactone) solution. Membranes were characterized by SEM, TEM, TGA, DSC, and ATR-FTIR. The mechanical properties and water contact angle were investigated as well. The antibacterial activity of the membranes were tested against *E. coli* and *S. aureus*. In addition, a real-time suitability observation of nisin-loaded PCL for food packaging was carried out.

## 2. Materials and Methods

### 2.1. Materials

In this study, poly(ε-caprolactone) (PCL, CAPA 6800) obtained from Solvay Interox Ltd. (Warrington, United Kingdom) with an average molar mass of M_w_ = 6.72 × 10^4^ g·mol^−1^ and with the dispersity of molar-mass ÐM = 1.56 was used. Dichloromethane (DCM) anhydrous, ≥99.8%, containing 50–150 ppm amylene stabilizer purchased from Sigma-Aldrich (Weinheim, German) and N, N-dimethylformamide (DMF) HPLC grade >99.7% (Alfa Aesar™, Karlsruhe, Germany), were used to prepare the polymer solutions. Nisin (N), from *Lactococcus lactis,* was purchased from Novazym Poland (Wielkopolska Centre of Advanced Technologies, Poznań, Poland). Casein-peptone lecithin polysorbate broth (CPLP broth) was obtained from Merck (Burlington, MA, USA). *Staphylococcus aureus* (CCM 4223) as a Gram-positive bacterium and *Escherichia coli* (CCM 3954) as a Gram-negative bacterium were purchased from the Czech Collection of Microorganisms from Masaryk University (Brno, Czech Republic).

Hayward kiwis (Actinia Deliciosa) imported from Italy, and cherry tomatoes (Solanum Lycopersicum), grown in Slovakia, were purchased from a commercial store chain in Bratislava, Slovakia.

### 2.2. Methods

#### 2.2.1. Preparation of Polymer Solutions for Electrospinning

PCL solutions were prepared in the DCM/DMF solvents mixture in a 1/1 volume ratio. The total concentrations of solutions are given in Table 1. PCL granules were weighted and delivered into the vials, and then the DCM was added. The storage solution was stirred on an IKA magnetic plate (Staufen im Breisgau, Germany) at an intensity of 650 rpm. After it was dissolved in DCM, the DMF was added to reach the required concentration. The storage solution was divided into six solutions, and nisin was added to the polymer solutions in the amount listed in Table 1. The total concentration of the resulting solutions is also listed in Table 1. 

The solution systems were ultrasonicated for 20 min just before measuring the viscosity and then before electrospinning to ensure the homogenous distribution of nisin in the solution.

#### 2.2.2. Viscosity of Solutions

The rheological measurements of plain PCL, as well as solutions of PCL with different concentrations of nisin (2, 6, 12, 25, and 50% m/m), were performed using a Rheometer AR2000 (TA Instruments, New Castle, DE, USA) at 25 °C in rotation mode using a DIN rotor Peltier cylinder system.

#### 2.2.3. Electrospinning

Electrospinning was carried out by a homemade experimental equipment setup in a horizontal spinning configuration without the possibility to control the ambient conditions (ambient temperature and humidity were 22 °C ± 1 °C and 57% ± 1%, respectively). A high-voltage power supplier (Spellman SL-150W, Bochum, Germany) was used to apply voltage with a positive polarity. The applied voltage was 17 kV. The solution was fed by a single syringe pump system, model NE-1000 (New Era Pump Systems, Inc., Farmingdale, NY, USA). The feeding rate was 1 mL per hour (mL/h). A needle with a 0.8 mm (21G) inner diameter was used to prepare membranes. The working distance between the top of the needle and the grounded collector (aluminum foil) was 14 cm. 

#### 2.2.4. Scanning Electron Microscopy

The morphology and average diameter of electrospun PCL fibers and fibrous composites were also observed through scanning electron microscopy (SEM, JSM Jeol 6610 microscope) at an accelerated voltage of 15 kV. The samples were sputtered with a thin layer of gold before observing. 

ImageJ software (freely available) was utilized to measure the average diameters of the fibers. The fibers’ average diameters and distributions were estimated statistically from 100 measured values from the three independently electrospun samples.

#### 2.2.5. Transmission Electron Microscopy

Morphological information was provided by a JEOL 1200FX transmission electron microscope (Jeol, Tokyo, Japan), which operated at 80 kV. The samples were collected directly during the spinning process and then also observed on copper grids.

#### 2.2.6. Attenuated Total Reflectance—Fourier Transform Infrared Spectroscopy

Fourier transform infrared (ATR-FTIR) spectra were recorded on a Nicolet 8700 spectrophotometer (Thermo Fisher Scientific, Madison, WI, USA). The spectra were taken in reflectance mode using the ATR (attenuated total reflectance) accessory and performed at 600–4000 cm^−1^ with the set resolution of 4 cm^−1^.

#### 2.2.7. Wettability

The water contact angle measurements of all investigated membranes were performed at room temperature. Water droplets were used with a drop volume of 30 µL. The camera Canon PowerShot SX130 (Tokyo, Japan) was used for taking images, and Image J software was used to evaluate the investigated material statistically.

#### 2.2.8. Mechanical Properties

The tensile properties were analyzed at 25 °C using a Dynamometer Instron 4301 (Instron Corporation, Norwood, MA, USA) according to standard ASTM D638. Seven electrospun membrane strips were cut for each measurement. The dimensions of the analyzed area of strip were 15 × 50 mm and a thickness of approximately 0.1 mm. The length of the cut strips was 100 mm for a more convenient manipulation/handling, and the sample gripping distance was constant for all measurements (50 mm). A testing rate of 1 mm·min^−1^ was used until a 0.5% deformation was achieved, and then the rate was immediately increased to 20 mm·min^−1^. Average tensile strength (σ_TS_), elongation at break (ε_B_), and Young’s modulus (E) values were calculated from the stress–strain dependencies.

#### 2.2.9. Thermogravimetric Analysis

The thermogravimetric analysis (TGA) of plain PCL and PCL with the addition of nisin was performed with a Mettler Toledo TGA/SDTA 851e (Mettler-Toledo, Columbus, OH, USA) instrument in parallel. Each measurement was performed at a temperature range of 25–600 °C under an inert atmosphere of nitrogen with a flow rate of 80 mL·min^−1^. The heat rate of the measures was 10 °C·min^−1^. The samples with a weight of 3–4 mg were placed in aluminum pans, and the instrument’s calibration was done according to the standard that was indium.

#### 2.2.10. Antibacterial Activity

The antibacterial activity of the electrospun plain PCL membranes and PCL fibrous membranes with the five different nisin concentrations was assessed with adherence to the procedure following ISO 22196:2011 for *S. aureus* and *E. coli* (ISO 22196, 2011). Suspensions of bacteria were prepared at concentrations between 2.5 × 10^5^ CFU·mL^−1^ and 10 × 10^5^ CFU·mL^−1^. The 40 mm × 40 mm samples and pieces of polyethylene film to cover them were treated by UV light for 30 min to sterilize the material just before the experiments. The 400 µL of suspension was applied to the surface of the plain PCL and nisin-loaded PCL membranes. The samples with suspension were covered with a polyethylene film. After a contact time of 24 h at 37 °C, the plain PCL and nisin-loaded PCL membranes were rinsed with 10 mL of casein–peptone lecithin polysorbate (CPLP) broth on a Petri dish, and the value for colony-forming cells (CFU·mL^−1^) was determined. The logarithm of reduction in the number of living and viable cells of tested bacteria (R) was calculated according to the Equation (1):

R = (Ut − U0) − (At − U0)(1)

where U_t_ is the mean for the common logarithm of the number of living bacteria, in (CFU·cm^−2^), recovered from the control samples (plain PCL) after 24 h, and A_t_ is the mean for the common logarithm of the number of living bacteria (CFU·cm^−2^), recovered from the test samples (PCL with different nisin concentrations) after 24 h. 

#### 2.2.11. Suitability Observation of Nisin-Loaded PCL for Food Packaging in Real-Time

The suitability of the investigated PCL fibrous samples with different concentrations of nisin for food packaging applications was assessed by comparing the storage resistance of unpacked and packed intact cherry tomatoes and precut kiwis in plain polycaprolactone and nisin-loaded PCL (PCL/12% N) fibrous membranes in a fridge in a controlled environment (relative humidity 75 ± 1% and temperature 4 ± 0.5 °C). The experiment took place until the first symptoms of spoilage appeared. Processes were loosely observed on individual pieces of tomatoes and kiwi (purchased from the market).

## 3. Results and Discussion

The development of a biodegradable food packaging on the base of electrospun fibrous membranes loaded with biocide nisin depends on the carriers’ properties of biologically active substances. In this study, the free-standing plain PCL and nisin-loaded PCL fibrous membranes with five different concentrations of nisin (listed in Table 1) were fabricated through electrospinning at the processing parameters reported in Section 2.2.3. The fibrous composites were prepared from the dispersion of nisin particles in a PCL solution. The binary solvent system was used to prepare the electrospun membranes. DCM was used as a solvent for PCL. DCM is used in many pharmaceutical applications as a process solvent. The Food and Drug Administration (FDA) has established residue tolerances for DCM [26]. As a cosolvent, DMF was selected due to its suitable conductivity, lower vapor pressure, and high dielectric constant [27]. DMF is widely used in the pharmaceutical industry as well, due to its solvating solid properties and organic reactions, which often cannot be achieved in less polar solvents [28]. PCL has already been electrospun from benign or greener solvents. However, a small average diameter, smoothness, and uniform fibers could not be achieved [29,30,31,32].

Electrospun nanofibrous mats were analyzed by scanning and transmission electron microscopy, attenuated total reflectance Fourier transform infrared spectroscopy, thermogravimetric analysis, and mechanical analysis. The antibacterial activity of the PCL and PCL membranes with all five concentrations of nisin was tested. In addition, the real-time suitability observation of nisin-loaded PCL for food packaging was carried out. The kiwi fruit and cherry tomatoes packed into the PCL/12% N membrane were observed for 20 and 28 days, respectively.

### 3.1. Analysis of the Morphology of the Investigated Samples

**Figure 1:**
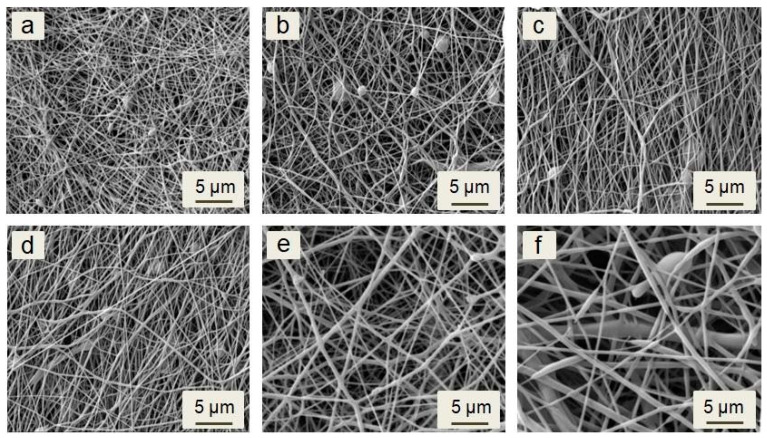
SEM micrograph of electrospun plain PCL (**a**), PCL/2% N (**b**), PCL/6% N (**c**), PCL/12% N (**d**), PCL/25% N (**e**), and PCL/50% N (**f**) fibrous membranes.

The morphology of all investigated electrospun membranes is shown on micrographs in Figure 1. The membranes contain randomly oriented continuous fibers. The beads are rarely observed. The imperfections of morphology could be caused by the impossibility of controlling the ambient environment parameters, such as temperature and humidity. According to previous studies, temperature and humidity could affect the morphology and fibers or beads formation [33,34,35,36]. 

Morphology of the fibers and their size distributions generally depends on the concentration and viscosity of solutions prepared for electrospinning [37,38]. Low viscosity could cause electrospraying or, in better cases, the formation of beads on the string instead of the continuous fibers. As the viscosity of the solution increases, the charged jet forms a straight shape in electrospinning, due to the increased chain entanglements. However, due to the increasing viscoelastic force, it can prevent the jet segments from being stretched by the constant coulombic force, thus resulting in a larger fiber diameter [39,40]. The viscosity is given by the concentration of the solution and molar mass of the used polymer and the solvent system. The solution in DCM has a lower viscosity than in chloroform, thus leading to a thinner fiber diameter in the DCM solvent. However, due to the low boiling point and high vapor pressure of DCM, un-uniform and irregular electrospun fibers were observed [39,41,42]. Therefore, a sufficient ratio of the binary solvent system such as DCM/DMF could solve the issue [29]. The viscosities of the prepared solutions and the fibers’ average diameters of all fibrous membranes are listed in Table 2. The plain PCL solution had the lowest viscosity in this study of 0.048 Pa·s. The fibers with an average diameter of 89 ± 50 nm were formed from the solution. The viscosity increased when increasing the peptide concentration, and the fibers’ average diameter increased. The maximal average diameter was observed for the sample PCL/50% N at 856 ± 423 nm, forming from the solution with a viscosity of 0.071 Pa·s.

**Figure 2:**
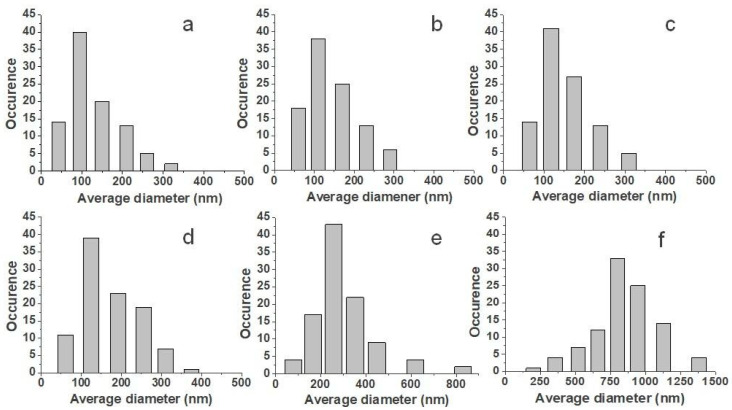
Distributions of fibers diameters: PCL (**a**), PCL/2% N (**b**), PCL/6% N (**c**), PCL/12% N (**d**), PCL/25% N (**e**), and PCL/50% N (**f**) fibrous membranes.

The distributions of fiber diameters are shown in Figure 2. With the increase of the nisin total final concentration and viscosity, the average diameter grew, and the fiber diameters distribution increased as well, as it is shown in Figure 2.

**Figure 3:**
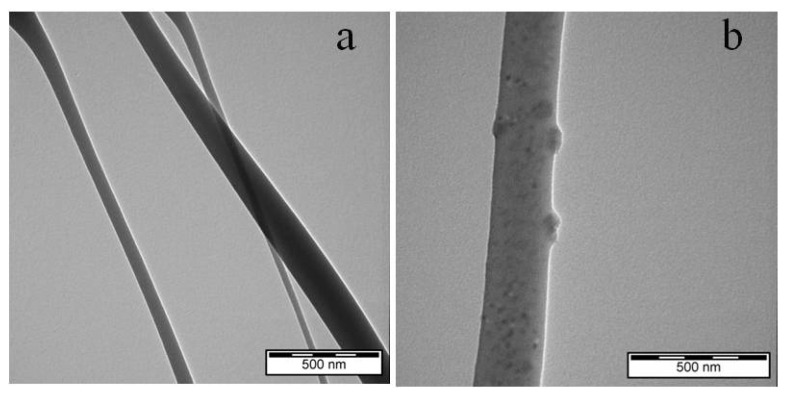
Representative TEM micrograph of electrospun plain PCL (**a**) and PCL/12% N (**b**) fibrous membranes.

A TEM study was conducted to provide a detailed view of the incorporation of nisin within electrospun membranes. The TEM micrograph is shown in Figure 3.

### 3.2. ATR-FTIR Analysis of PCL and Fibrous Composites PCL/N

**Figure 4:**
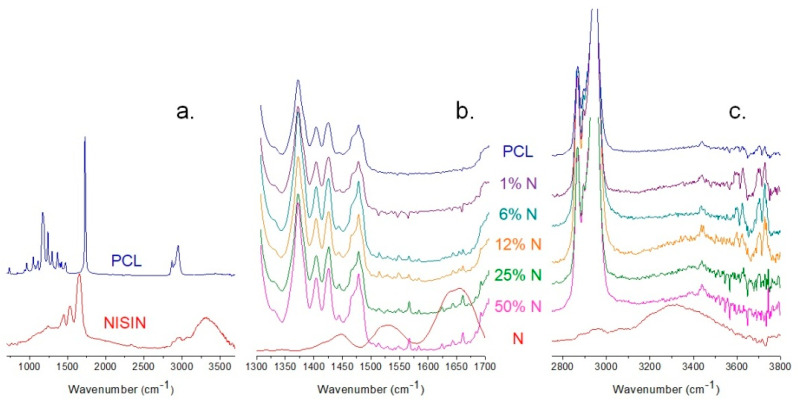
The infrared spectra of (**a**) plain PCL and nisin, and comparison of plain samples to the composites PCL/nisin samples in detail in regions (**b**) 1300–1700 cm^−1^ and (**c**) 2750–3800 cm^−1^.

ATR-FTIR spectroscopy was employed to identify the characterized materials’ chemical structure and interactions between the components of the PCL/N nanofibrous membranes. The infrared spectra are shown in Figure 4. All samples were analyzed in the region 600–4000 cm^−1^. 

The spectra of the individual components are shown in Figure 4a. The spectrum of PCL shows peaks at 2841 and 2931 cm^−1^ corresponding to the C-H symmetric and antisymmetric stretching, and a strong signal at 1718 cm^−1^ appertaining to the C=O of the ester group. The spectrum shows nisin contains O-H and a N-H axial stretching at 3316 cm^−1^, and a stretching vibration of the C-H bond appeared at 2965 cm^−1^. The characteristic absorption of the C=O group and the NH_3_^+^ band at 1650 and 1525 cm^−1^ entirely disappeared at the combination of PCL and nisin due to the hydrogen bonding creation (Figure 4b) (Jiang et al., 2020). The decrease in the intensity and the shift of the broad peak of O–H stretching from 3310 to 3425 cm^−1^ indicate the involvement of the O–H and N-H groups in the formation of hydrogen bonding between the nisin and PCL (Figure 4c). However, no significant changes were observed in other characteristic signals of the PCL/N composites.

### 3.3. Analysis of Wettability

Measuring the water contact angle is a key technique to determine the surface wettability of membranes by a liquid. The static water contact angle was measured to determine the wettability of plain PCL, PCL/2% N, PCL/6% N, PCL/12% N, PCL/25% N, and PCL/50% N membranes. The water contact angle was evaluated based on the nisin concentration in the PCL membrane. The results are listed in Table 3.

The contact angle of PCL was 100° ± 5°; however, with an increasing concentration of nisin, the contact angle increased very slightly, but the differences were within the margin of error.

Nisin has been used for decades as a food preservative. Due to its amphiphilic nature, it can adsorb on both types of surfaces, hydrophobic and hydrophilic, and retain a stable activity in the adsorbed state [13]. In this case, when nisin was incorporated into the structure of the fibers, the surface of fibrous composites remained hydrophobic.

### 3.4. Mechanical Analysis

**Figure 5:**
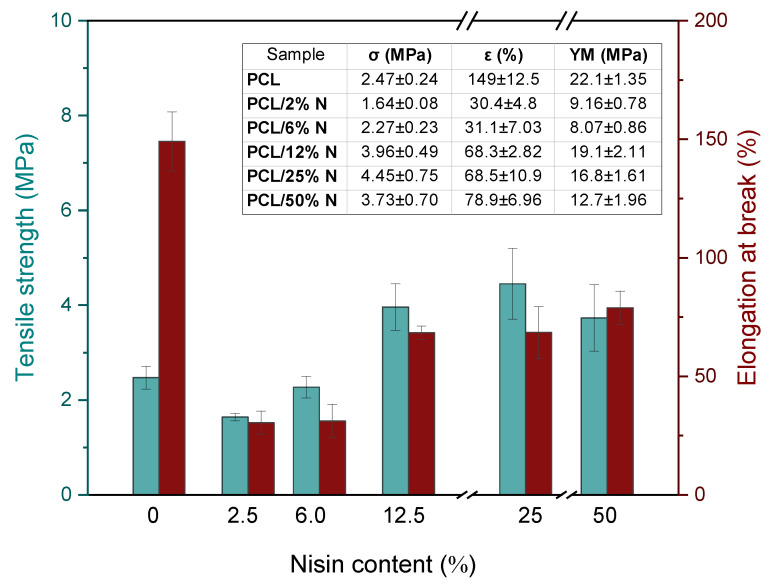
Changes in mechanical properties such as Young´s modulus (YM), tensile strength (σ) and elongation at break (ε) for PCL membrane strips with different nisin concentrations varied from 0 to 50%.

The mechanical properties of PCL and its composites containing nisin are summarized in Figure 5. As it is clearly seen from the inset table, the initial low nisin concentration leads to a significant drop in elongation at the break due to the preferable interaction of nisin particles with each other, resulting in the forming of bulkier bundles. This hypothesis is supported by the nature of nisin bearing numerous representations of polar amino and hydroxyl functional groups that can prefer mutual interaction and de facto act as cross-linkers. Initial lower nisin amount caused a slight decrease in the tensile strength values in comparison to neat PCL due to a worsening of the compatibility and disintegration of the initial arrangement of the polymer chain in the material. With an increasing nisin concentration, a gradual increase in both the tensile strength and elongation at break occurred due to more numerous interactions within the composite material between the polymer matrix and the filler particles. This phenomenon was also supported by the comparison of the Young´s modulus values, which showed the same tendency of the initial drop at lower nisin concentration, up to 6%, followed by a sharp increase to the values close to the ones of the neat PCL matrix.

**Figure 6:**
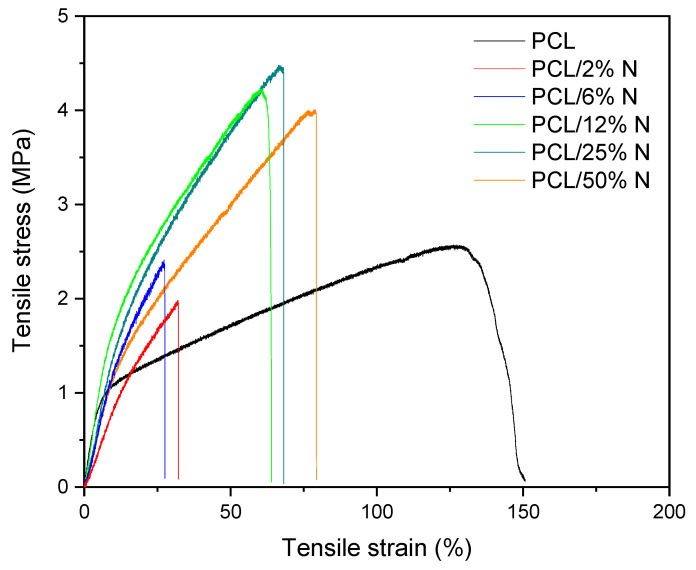
Stress–strain curves for plain electrospun PCL and PCL mats with different concentrations of nisin solution varied from 0 to 50%.

On the other hand, a possible explanation of this behavior can be ascribed to the percolation theory; at low concentrations, the particles are isolated in the polymer, their presence leading to a significant worsening of the mechanical properties of composites. When percolation is reached, at the percolation threshold, there is a sharp change in properties due to the formation of a 3D network as a result of the mutual interactions of the particles. According to this hypothesis, the stress–strain curves summarized in Figure 6 show a typical tensile behavior for a composite filled with a reinforced filler. We reported this in our earlier study focused on a composite filled with keratin [43]. In the case of a nisin composite, we observed a significantly brittle behavior already at the lowest concentration, accompanied by the disappearance of yield strength due to a pronounced stiffness in the material. The addition of a higher nisin content led to a progressive tensile strength increase, accompanied by the sharp decline of elongation at break as a consequence of the insufficient mobility of the PCL chains in the amorphous region. The presented nisin particles reduced the chain movement because they act as a bulky barrier for relaxation processes leading to limited options for straightening the PCL chains under tension.

### 3.5. Thermogravimetry Analysis

**Figure 7:**
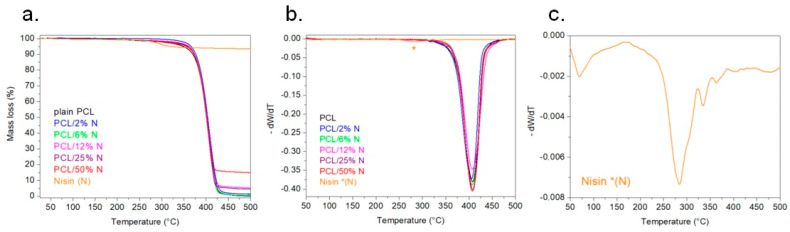
Nonisothermal TGA records of plain PCL and PCL with the addition of nisin under a nitrogen atmosphere (**a**), graphs of 1st derivation of TGA (**b**), and detail of 1st derivation of nisin TGA (**c**).

The thermal decomposition curves of obtained membranes indicated a single mass-loss step. Figure 7 depicts the thermal stability of the investigated samples. The thermal stability was studied in an inert atmosphere from room temperature to 500 °C using the TGA instrument. From the TGA records, it can be seen that all samples had a very similar course. In terms of thermal stability, the addition of nisin did not influence PCL at all. The decomposition of all the PCL/N samples started around 380 °C. However, the remaining weight after the deterioration step differed depending on the membrane’s composition. A higher addition of nisin meant a higher amount of material residue at that temperature. The literature shows that the mass loss of nisin around the temperature of 600 °C is close to 10% wt [44]. From Figure 7, the highest amount of residue of the sample was for the sample with the highest addition of nisin. The residue of that sample (PCL/50% N) was around 14% wt at 500 °C. The first-order derivative (Figure 7b) confirmed that the temperature of decomposition of PCL and PCL/N was about 400 °C. Moreover, Figure 7c shows the first derivative of nisin TGA in detail.

### 3.6. Testing for Antibacterial Activity

A wide range of enteric pathogens such as the enterovirulent *E. coli* and *S. aureus* can be transmitted via food. These bacteria strains can be responsible for reducing the shelf life and acceptability of fresh produce. It is not surprising that enteric pathogens can contaminate agricultural produce and cause illness due to the widespread utilization of human and animal fecal waste in agricultural practice [45]. Therefore, the antibacterial activity of electrospun plain PCL and nisin-loaded PCL fibrous membranes: PCL/2% N, PCL/6% N, PCL/12% N, PCL/25% N, and PCL/50% N were investigated with adherence to the procedure stipulated by the ISO 22196:2011 for *S. aureus* as Gram-positive rods and *E. coli* as Gram-negative cocci, respectively. 

The bacterial viability on the samples was investigated after 24 h of contact with the tested membranes. The antibacterial activity, as well as the reduction of viability, is listed in Table 4.

This experiment showed that the growth of bacteria on the PCL membranes at different nisin concentrations was reduced for both tested bacteria strains. Two percent of nisin was enough to achieve a reduction of more than 50% of *E. coli* strain and more than 60% *S. aureus* strain growth after 24 h of contact. With the increase in nisin concentration, bacterial growth is further reduced. PCL with 12–50 wt% of nisin concentrations resulted in a reduction of more than 90% in *E. coli* and *S. aureus* cells. 

The results of the studies showed that nisin as an effective inhibitor of the selected bacteria growth.

### 3.7. Suitability Observation of Nisin-Loaded PCL for Food Packaging

Since the nonwoven fabric of PCL/12% N demonstrated an inhibition of more than 90% of cell viability of both tested specimens of bacteria, it was chosen as a representative sample for the potential application for food packaging. The mat of plain PCL was used for the comparison study. The results were compared to the same food storage in a plastic container closed with a lid. The tomatoes and sliced halves of kiwi were placed into plastic containers. Three types of packing were applied:

- Tomatoes and kiwi were freely placed into an open plastic container;
- Tomatoes and kiwi were covered by nonwoven fabric mate of plain PCL membranes before putting them into an open container and;
- Tomatoes and kiwi were covered by a PCL/12% N nonwoven fabric mat before they were placed in an open container.

**Figure 8:**
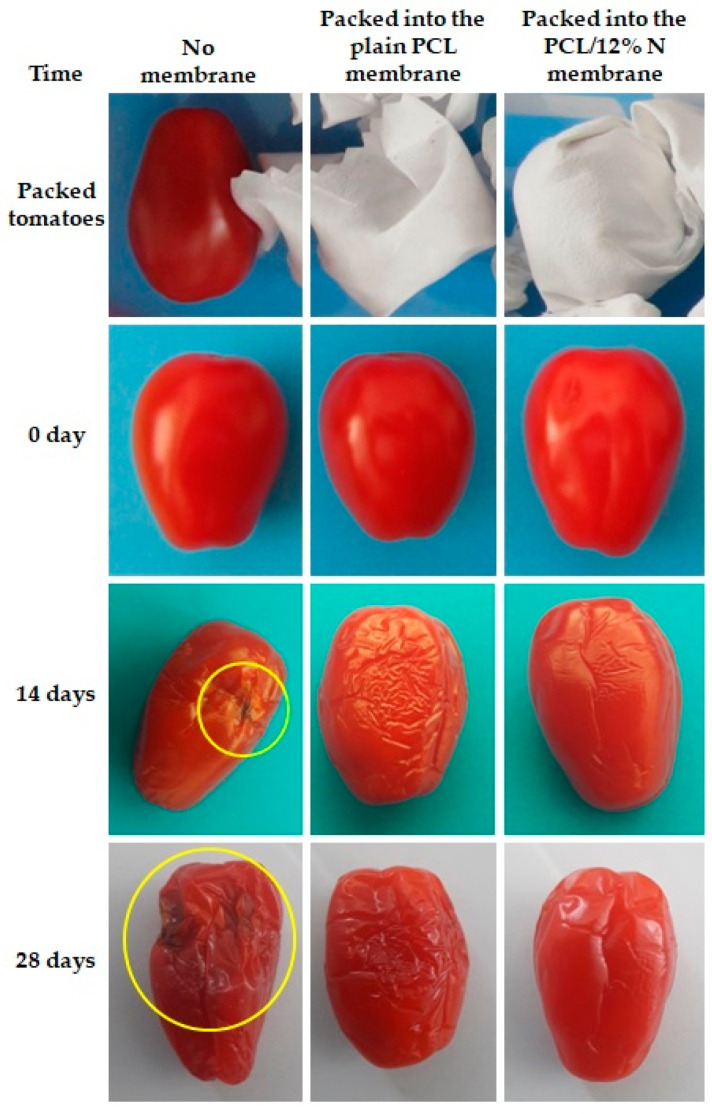
Observational experiments over time with tomatoes for suitability of fibers-based food packaging. The yellow circle highlights the rot.

**Figure 9:**
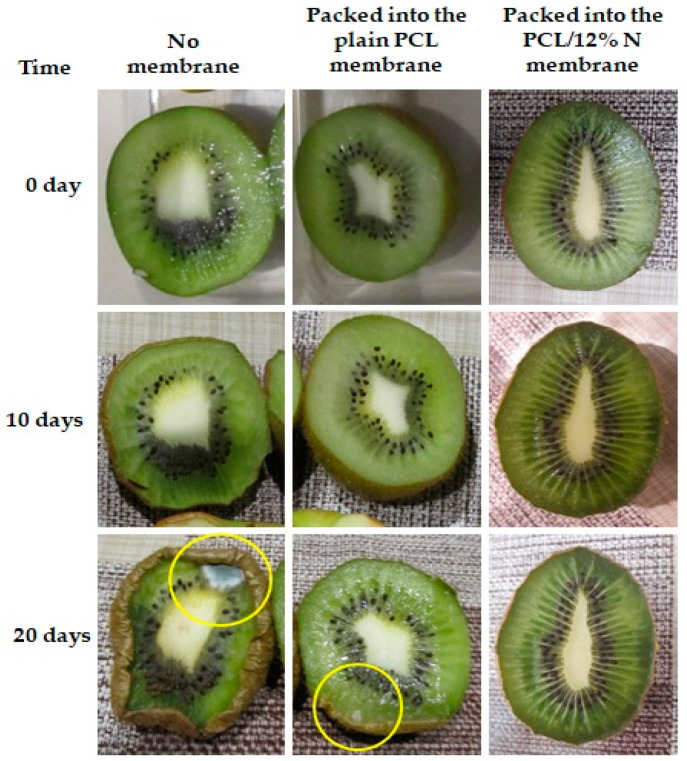
Observational experiments over time with kiwi for suitability of fibers-based food packaging. The yellow circle highlights the rot.

Then, the samples in the open plastic container were kept in a controlled environment, in a refrigerator at 4 °C and at 78% humidity. Digital photographs were systematically taken to follow the changes in cherry tomatoes (Figure 8) and kiwi (Figure 9). The experimental study lasted 28 days for cherry tomatoes and 20 days for kiwi.

The first symptoms of tomatoes spoilage, regardless of the packaging, were observed after 14 days of storage. However, it is worth noticing that fruits stored in nonwoven fabric loaded with nisin demonstrated the lowest degree of spoilage compared to fruits packaged in open plastic containers and plain PCL fibrous mats. After 28 days of storage, spoilage was observed mostly on fruits stored in a plastic container and plain PCL mat; meanwhile, fruits packed with the PCL/12% N membrane kept a freshness comparable to that of the fruits after 14 days.

Kiwi fruits are commonly known as fruit with a high susceptibility for spoilage; for this reason, the period of observation was shortened and divided into two, namely after 10 and 20 days. The tendency of kiwi fruit to spoil was similar to that of tomatoes. After 10 days, the first symptoms of spoilage were observed; however, the degree of spoilage was the lowest for fruits stored in nonwoven mats of PCL loaded with 12% of nisin. It has to be highlighted that after 20 days, kiwi fruits were still fresh and unspoiled when packed into nonwoven fabrics of polycaprolactone loaded with nisin. The prolongation of fresh fruits packed with plain PCL fibrous mats was probably caused by the porous electrospun mats’ easy air and water permeability, as discussed before [46]. Based on the obtained results devoted to the impact of the type of packaging on the freshness of selected fruits, it can be concluded that the addition of nisin plays a role as an active factor in facilitating the prolongation of freshness by inhibiting the spoilage process and simultaneously enhancing the shelf life of fruits. 

Electrospinning methods have become more and more popular because they are considered innovative packaging approaches in the biomedical sector and food industry [47,48,49]. Packaging made of fibrous mats undoubtedly facilitates oxygen/carbon dioxide permeation, which is very much required to store vegetables and fruits [50]. Additionally, the presence of nisin, which was chosen as a representative among other antioxidant and antimicrobial compounds (essential oils, curcumin, α tocopherol, vitamins, phenolic-rich extracts from plants, etc.), embedded in the polymer matrix of packaging prolongs the food freshness [48,51]. Jin et al. [52] prepared a film of pectin/PLA blend with nisin via an extrusion process. This composite was efficient in inhibiting *Listeria monocytogenes*, both in an in vitro assay and in food samples. Wu et al. [53] evaluated a biodegradable membrane from oxidized cellulose incorporated with nisin on *Alicyclobacillus acidoterrestris* DSM 3922T and found it to have long-term antimicrobial activity. PVA electrospun nanomats loaded with curcumin/nisin were actively used as packaging material for trout fillets and fish *Oncorhynchus mykiss* flesh [54,55]. Composite of chitosan/PVA loaded with nisin caused inhibition of total viable bacteria, psychrophilic bacteria, yeast, and mold growth in the flesh, leading to a prolonged freshness in fish and fish products. There are a lot of articles in the literature concerned with the prolongation of fruits’ and vegetables’ freshness [56,57,58]; however, according to our knowledge, the topic devoted to nanofibrous packaging made by the electrospinning method for this purpose has risen dramatically [59]. Resende et al. [60] demonstrated that a chitosan/cellulose nanofibril coating reduced respiration and oxygen diffusion and delayed strawberry oxidation. Composites containing more nanofibril exhibited a higher barrier against oxygen. Wen et al. [61] found that strawberries packed in a nanofibrous polyvinylalcohol (PVA)/cinnamon essential oil (EO)/βcyclodextrin film kept their freshness much longer compared to control fruits and demonstrated no decay even at day 6. Moreover, no changes in their flavor were noticed, extending the same shelf life of stored strawberries.

Li et al. [62] reported that the coaxial electrospun eugenol-loaded core-sheath polyvinylpyrrolidone/shellac fibrous films prolonged the fruit quality and extended the shelf life of strawberries. Min et al. [63] prepared packages made of polyvinyl alcohol film incorporated with thyme-essential-oil-loaded polylactide electrospun nanofibers to store strawberries. They also observed that the shelf life of strawberries during storage was significantly extended.

The usefulness of electrospun phospholipid/PEO nanofibers containing encapsulated cinnamaldehyde/hydroxypropyl-β-cyclodextrin inclusion complex as a packaging material to preserve fresh-cut cucumber was examined by Li et al. [64]. They found that prepared nanofibrous mats exhibited an inhibitory effect against *L. monocytogenes* without a considerable impact on tested vegetables’ sensory parameters and surface color.

In the study of Ansarifar and Moradinezhad [65], the authors prepared by electrospinning technique an active packaging with encapsulated thyme essential oil in a zein nanofiber mat; they found that during 15 days of storage at 4 °C, strawberries stored in these packaging films significantly reduced the total bacterial counts, fungi, and yeast. Similar results were obtained by Zhang et al. [66]. They stored strawberries in biodegradable poly(lactide-co-glycolide) nanofibers loaded with thymol and revealed that such fibrous mats might successfully prevent the growth of fungi, yeast, and bacteria and prolong the shelf life of fruits.

Recently, Fonseca et al. examined the thermal resistance, antimicrobial and antioxidant activity of *E. coli*, *S. Typhimurium*, *S. aureus*, and *L. monocytogenes* in the presence of encapsulated carvacrol in starch-based electrospun nanofibers. A moderate antimicrobial activity against all the tested pathogens was shown. However, the obtained results indicated that the starch/carvacrol nanofibers are a potential antimicrobial and antioxidant agent for application in the food sector [67].

## 4. Conclusions

Regardless of preparation, biodegradable active packages are an exciting alternative to conventional packaging materials. They are safe for the environment and can reduce food waste by extending the shelf life. Nonwoven fabric packages have advantages over typical plastic containers. The permeation of gases such as carbon dioxide, oxygen, or others is facilitated due to the high porosity of fibrous membranes. Additionally, incorporating into a polymer fibrous matrix a nontoxic active agent such as nisin definitely improves the antispoilage properties of these packages, which represents an excellent alternative for the common problems appearing during storage, especially in summer. The preparation of such fibrous active packages possesses another economic and environmental advantage. Due to the high porosity, the amount of material used for preparation is much lower than in traditional methods. The only disadvantage we can expect is that the biodegradability of PCL may be prolonged in compost conditions after use, because, by definition, biodegradability proceeds via the action of microorganisms able to convert polymer by their enzymes to water and carbon dioxide.

For this reason, the presence of antimicrobial agents such as nisin may inhibit the biodegradation process. However, more detailed research on it is required. Even if environmental and economic benefits come from the preparation of totally environmentally friendly packaging, the benefits of combining biodegradable polymers and naturally occurring nisin are uncontested.

## Figures and Tables

**Table 1:** Solutions prepared for electrospinning of PCL and PCL/nisin fibers (N—nisin, DCM, DMF—used solvents).

Sample	PCL (g)	Nisin (g)	DCM (mL)	DMF (mL)	Total Concentration (%) (m/V)
PCL	1.5	-	5	5	15.00
PCL/2% N	1.5	0.03	5	5	15.30
PCL/6% N	1.5	0.09	5	5	15.90
PCL/12%N	1.5	0.18	5	5	16.80
PCL/25% N	1.5	0.38	5	5	18.80
PCL/50% N	1.5	0.75	5	5	22.50

**Table 2:** The values of the viscosity of solutions, average diameters (avg. dia.) of the tested electrospun fibers PCL and PCL/N electrospun membranes, and standard deviation (S_d_) of five replicates.

Sample	Viscosity (Pa·s)	Avg. dia ± S_d_ (nm)
PCL	0.048	89 ± 50
PCL/2% N	0.049	106 ± 47
PCL/6% N	0.050	118 ± 53
PCL/12% N	0.051	123 ± 43
PCL/25% N	0.057	258 ± 122
PCL/50% N	0.071	856 ± 423

**Table 3:** Contact angle values of investigated samples.

Sample	PCL	PCL/2% N	PCL/6% N	PCL/12% N	PCL/25% N	PCL/50% N
Contact angle (°)	100 ± 5	99 ± 7	100 ± 6	101 ± 5	103 ± 4	104 ± 5

**Table 4:** The contact method tested both antibody activity and efficiency (quantitative) *E. coli* CCM 3988 and *S. aureus* CCM 3953. The concentration of bacteria in the test inoculum was 500,000 CFU·mL^−1^.

Tested Microorganism	Amount of Nisin (%)	Number of Bacteria Recovered at 24 h of Contact Time (CFU *·mL^−1^)	Log. of Number of Bacteria Recovered at 24 h Contact Time (CFU *·mL^−1^)	Antibacterial Activity R	Reduction (%)
*E. Coli* CCM 3988 (G−)	-	2,300,000 ± 150,000	6.36 ± 0.41	-	-
	2	1,100,000 ± 40,000	6.04 ± 0.22	0.32 ± 0.19	52.17 ± 3.63
	6	450,000 ± 26,000	5.65 ± 0.33	0.71 ± 0.08	80.43 ± 5.77
	12	200,000 ± 12,000	5.30 ± 0.32	1.06 ± 0.09	91.30 ± 6.00
	25	130,000 ± 9000	5.11 ± 0.35	1.25 ± 0.06	94.35 ± 6.92
	50	120,000 ± 7700	5.08 ± 0.33	1.28 ± 0.08	94.78 ± 6.42
*S. Aureus* CCM 3953 (G+)	-	2,100,000 ± 112,000	6.32 ± 0.34	-	-
	2	800,000 ± 38,000	5.90 ± 0.28	0.42 ± 0.06	61.90 ± 4.75
	6	300,000 ± 17,500	5.48 ± 0.32	0.84 ± 0.02	85.71 ± 5.83
	12	180,000 ± 9000	5.26 ± 0.26	1.06 ± 0.08	91.43 ± 5.00
	25	130,000 ± 7000	5.11 ± 0.28	1.21 ± 0.06	94.35 ± 5.38
	50	120,000 ± 5500	5.08 ± 0.23	1.24 ± 0.11	94.78 ± 4.58

* Colony-forming unit (CFU), (G−)—Gram-negative bacteria, (G+)—Gram-positive bacteria.

## Data Availability

Not applicable.
